# CD38 Correlates with an Immunosuppressive Treg Phenotype in Lupus-Prone Mice

**DOI:** 10.3390/ijms222111977

**Published:** 2021-11-05

**Authors:** Jocelyn C. Pérez-Lara, Enrique Espinosa, Leopoldo Santos-Argumedo, Héctor Romero-Ramírez, Gabriela López-Herrera, Fabio García-García, Claudia Sandoval-Montes, Vianney Ortiz-Navarrete, Mónica Flores-Muñoz, Juan C. Rodríguez-Alba

**Affiliations:** 1 Programa de Doctorado en Ciencias de la Salud, Universidad Veracruzana, Xalapa 91050, Mexico; jocperez@uv.mx; 2 Laboratorio de Inmunología Integrativa, Instituto Nacional de Enfermedades Respiratorias, Ciudad de México 14080, Mexico; hector.enrique.espinosa@gmail.com; 3 Centro de Investigación y de Estudios Avanzados-IPN, Departamento de Biomedicina Molecular, Ciudad de México 11340, Mexico; lesantos@cinvestav.mx (L.S.-A.); hromero@cinvestav.mx (H.R.-R.); vortiz@cinvestav.mx (V.O.-N.); 4 Unidad de Investigación en Inmunodeficiencias, Instituto Nacional de Pediatría-SSa, Ciudad de México 04530, Mexico; lohegabyqbp@gmail.com; 5 Laboratorio de Biología del Sueño, Instituto de Ciencias de la Salud, Universidad Veracruzana, Xalapa 91050, Mexico; fgarcia@uv.mx; 6 Laboratorio de Inmunología, Escuela Nacional de Ciencias Biológicas, IPN, Ciudad de México 11340, Mexico; claudiaqfb@yahoo.com.mx; 7 Laboratorio de Medicina Traslacional, Instituto de Ciencias de la Salud, Universidad Veracruzana, Xalapa 91050, Mexico; moflores@uv.mx; 8 Unidad de Citometría de Flujo, Instituto de Ciencias de la Salud, Universidad Veracruzana, Xalapa 91050, Mexico

**Keywords:** CD38, regulatory T-cells, systemic lupus erythematosus, immunosuppressive, lupus-prone mice, IFN-γ, IL-10

## Abstract

CD38 is a transmembrane glycoprotein expressed by T-cells. It has been reported that patients with systemic lupus erythematosus (SLE) showed increased CD38^+^CD25^+^ T-cells correlating with immune activation and clinical signs. Contrariwise, CD38 deficiency in murine models has shown enhanced autoimmunity development. Recent studies have suggested that CD38^+^ regulatory T-cells are more suppressive than CD38^−^ regulatory T-cells. Thus, we have suggested that CD38 overexpression in SLE patients could play a role in regulating immune activation cells instead of enhancing it. This study found a correlation between CD38 with FoxP3 expression and immunosuppressive molecules (CD69, IL-10, CTLA-4, and PD-1) in T-cells from lupus-prone mice (B6.MRL-Fas^lpr^/J). Additionally, B6.MRL-Fas^lpr^/J mice showed a decreased proportion of CD38^+^ Treg cells regarding wild-type mice (WT). Furthermore, Regulatory T-Cells (Treg cells) from *CD38-/-* mice showed impairment in expressing immunosuppressive molecules and proliferation after stimulation through the T-cell receptor (TCR). Finally, we demonstrated an increased ratio of IFN-γ/IL-10 secretion in *CD38-/-* splenocytes stimulated with anti-CD3 compared with the WT. Altogether, our data suggest that CD38 represents an element in maintaining activated and proliferative Treg cells. Consequently, CD38 could have a crucial role in immune tolerance, preventing SLE development through Treg cells.

## 1. Introduction

Systemic lupus erythematosus (SLE) is a chronic, multiorgan, systemic autoimmune disease that affects nearly every tissue and organ system [1]. As with other autoimmune diseases, SLE more frequently affects women than men, with female to male ratios exceeding 9:1 [2]. The etiological mechanism of SLE remains unclear. However, genetic, hormonal, and environmental factors and a loss of tolerance have been associated with disease development [3,4]. Preliminary studies of genetic factors have demonstrated that European–American and Icelandic populations have an SLE susceptibility locus on the human region 4p15 [5,6]. Located within the 4p15 region is the gene encoding CD38 [7], a multifunctional transmembrane glycoprotein that has both enzymatic and receptor properties [8,9]. As an enzyme, CD38 can convert nicotinamide adenine dinucleotide (NAD+) into cyclic adenosine diphosphate ribose (cADPR) [10]. Otherwise, activation of CD38 receptor activity induces several cell responses such as activation, migration, proliferation, differentiation, and apoptosis in different immune cell types [11,12,13,14]. Previous studies have shown increased levels of CD38 expressing T and B cells in SLE patients compared to healthy controls [15,16,17,18]. Remarkably, Pavón et al. reported higher levels of CD38 expression in CD25^+^ T-cells from SLE patients than in healthy controls [19], suggesting that CD38 expression is derived from chronic immune activation correlating with disease severity. In contradiction with these previous reports, some authors have suggested that CD38 plays a role in preventing autoimmunity. Several studies have found that CD38 absence in murine autoimmune models enhances illness severity [20,21]. We have demonstrated that CD38 deficiency in a non-autoimmune-susceptible strain (*CD38-/-*) developed some autoimmune characteristics at old age [22], suggesting that CD38 could play a role in maintaining immune homeostasis. Furthermore, CD38 was upregulated in regulatory B cells (Bregs), and CD38 cross-linking with an agonistic antibody increased the frequency of Breg cells and IL-10 production [22]. Altogether, it seems that the CD38 receptor can play an essential role in preventing autoimmunity mechanisms, mainly through the regulation of the immune system.

Regulatory T-cells (Treg cells) play a crucial role in regulating the immune response, maintaining peripheral tolerance, and controlling the development of several autoimmune diseases, such as SLE [23]. Treg cells are identified by CD25 and FoxP3 expression in CD4^+^ T-cells from mice, while CD4^+^CD25^hi^CD127^low^ T-cells have been widely accepted to identify Treg cells in humans [24,25]. Additional markers have been described to select functional Treg cells, highlighting immunosuppressive molecules such as CTLA-4, IL-10, CD69, and PD-1 [26,27,28,29]. A further vital mechanism to maintain immune tolerance is Treg proliferation, which is required to expand these suppressive cells and avoid autoimmune diseases [30].

In the last few years, an increasing number of studies in patients with multiple myeloma have found that CD38^+^ Treg cells are more suppressive than CD38^−^ Treg cells and antagonist antibodies can block their inhibitory activity [31,32]. Despite this, the role of CD38 in SLE disease has not yet been established. Therefore, we hypothesized that CD38 expression in T-cells could play a role in immune regulation instead of immune activation. To evaluate this, we correlated CD38 expression in T-cells with FoxP3 expression and immunosuppressive markers in lupus-prone mice. Furthermore, we assessed immunosuppressive molecules and proliferative capability in Treg cells from *CD38-/-* mice. Finally, we defined that CD38 deficiency promoted a dysregulated IFN-γ/IL-10 cytokine secretion when splenocytes were stimulated with anti-CD3/CD28 + rhIL-2. We found that CD38 expression is essential for maintaining the immunoregulatory phenotype and expanding Treg cells from lupus-prone mice.

## 2. Results

### 2.1. CD38 Expression Positively Correlates with FoxP3 Expression

**Figure 1:**
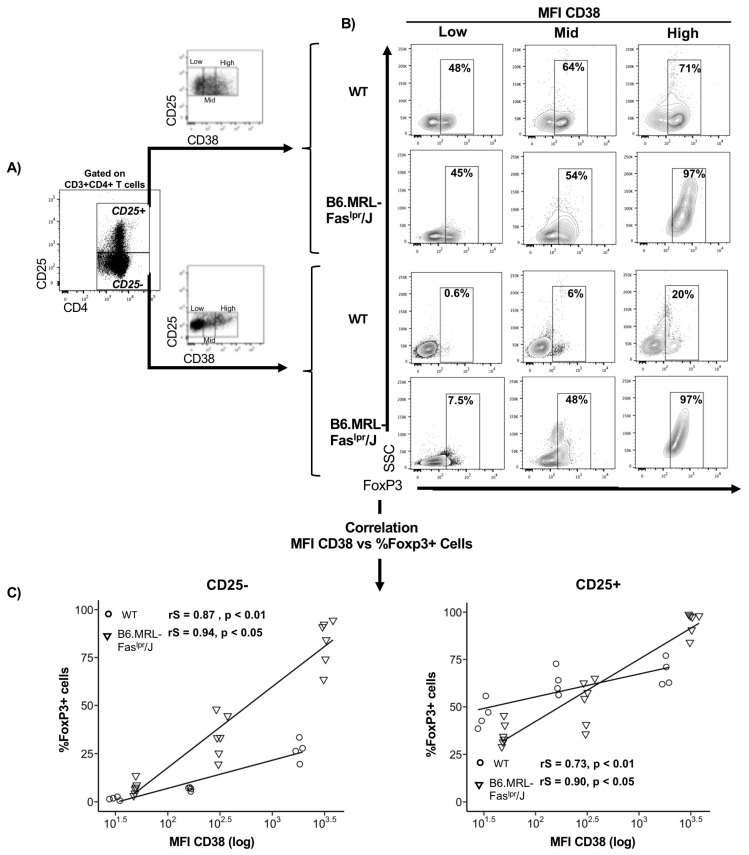
The level of CD38 correlates with FoxP3 frequency in T CD25^+^ and CD25^−^ T-cells. Splenocytes were stained for cell surface expression of CD3, CD4, CD25, and CD38. The stained cells were fixed and stained intracellularly for FoxP3. (**A**) Splenocytes from wild type (WT) and B6.MRL-Fas^lpr^/J were gated on CD3^+^CD4^+^ cells and analyzed for CD25 expression. Then, CD3^+^CD4^+^CD25^+^ or CD25^−^ cells were gated on CD38^low^, CD38^mid^, or CD38^high^, according to CD38 expression; (**B**) counter plots showing FoxP3+ cells frequency in CD38^low^, CD38^mid^, or CD38^high^ subsets among CD25^+^ and CD25^−^ T-cells from WT and B6.MRL-Fas^lpr^/J; (**C**) correlation plots comparing MFI of CD38 to FoxP3^+^ cells within either CD4^+^CD25^−^ T-cells (top) or CD4^+^CD25^+^ T-cells (bottom). Symbols represenT-cells from WT (*n* = 4) (open symbols) and B6.MRL-Fas^lpr^/J (*n* = 6) (filled symbols). The inset lines are the product of linear regression analysis. Correlations were calculated by the Spearman method.

To assess the regulatory role of CD38 in T-cells from a lupus-prone mouse (B6.MRL-Fas^lpr^/J), we examined FoxP3 expression in three different subsets of CD3^+^CD4^+^CD25^+^ and CD3^+^CD4^+^CD25^−^ T-cells, gated according to CD38 expression. As shown in Figure 1A, we identified CD38^low^, CD38^mid^, and CD38^high^ cells within CD4^+^CD25^+^ and CD4^+^CD25^−^ T-cell subsets from WT and B6.MRL-Fas^lpr^/J mice. We then analyzed the frequency of FoxP3 expressing cells in each subset (Figure 1B). As shown in Figure 1B, we noted an increased amount of Foxp3+ cells when CD38 increased its expression in CD25^+^ and CD25^−^ cells from WT and B6.MRL-Fas^lpr^/J mice. Thus, we assessed the relationship between the surface expression of CD38 and the transcription factor, FoxP3. We plotted the mean fluorescence intensity (MFI) of CD38^low^, CD38^mid^, and CD38^high^ against the number of cells positive for FoxP3 from CD25^+^ and CD25^−^ cells in WT and B6.MRL-Fas^lpr^/J mice. Using Spearman correlation analysis, we displayed a positive correlation between CD38 and frequency of Foxp3 cells among CD4^+^CD25^−^ from WT (rS = 0.87, *p* < 0.01) and B6.MRL-Fas^lpr^/J mice (rS = 0.94, *p* < 0.05) (Figure 1C). In the same way, CD4+CD25+ cells showed a positive correlation between CD38 and Foxp3 cells in WT (rS = 0.73, *p* < 0.01) and B6.MRL-Fas^lpr^/J mice (rS = 0.90, *p* < 0.05) (Figure 1C). Additionally, correlation plots showed higher CD38 expression in CD25^+^ and CD25^−^ cells from B6.MRL-Fas^lpr^/J than WT mice. These results suggest that CD38 expression may identify subsets with regulatory characteristics.

### 2.2. CD38 Expression Positively Correlates with Immunosuppressive Molecules

**Figure 2:**
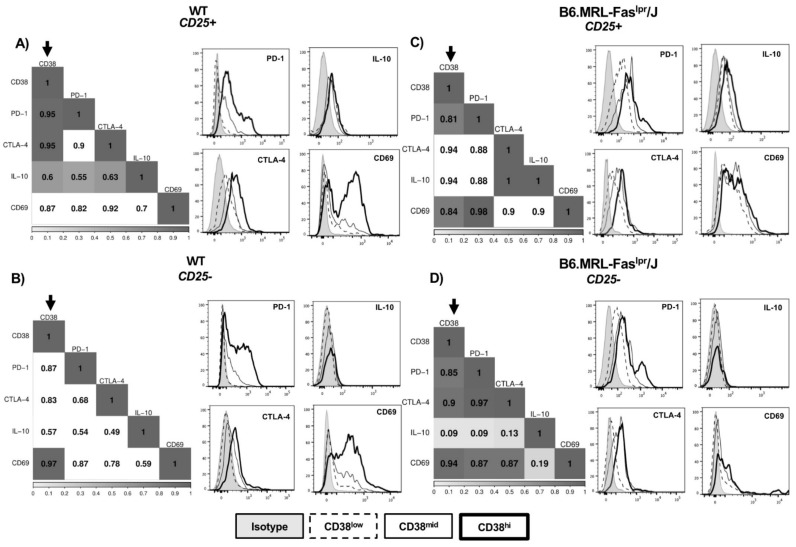
CD38 correlates with immunosuppressive molecules. Correlation matrix and histograms of PD-1, CTLA-4, IL-10, and CD69 expression within CD38^low^ (dashed line), CD38^mid^ (black line), and CD38^high^ (heavy line) subsets in (**A**) WT CD4^+^CD25^+^ T-cells (*n* = 6); (**B**) WT CD4^+^CD25^−^ cells (*n* = 6); (**C**) B6.MRL-Fas^lpr^/J CD4^+^CD25^+^ T-cells (*n* = 6); and (**D**) B6.MRL-Fas^lpr^/J CD4^+^CD25^−^ T-cells (*n* = 6). Darker squares into correlation matrices point out a correlation close to 1. Shaded squares indicate correlations with a *p* < 0.005, while blank squares show non-significative correlations.

To understand the possible regulatory relevance of CD38 expression in CD4^+^CD25^+^ and CD4^+^CD25^−^ T-cells, we assessed by flow cytometry the MFI of CD69, IL-10, CTLA-4, and PD-1 from CD38^low^, CD38^mid^, and CD38^high^ subsets from WT (Figure 2A,B) and B6.MRL-Fas^lpr^/J (Figure 2C,D). Then, we developed a Spearman correlation matrix including MFI values from each CD38 subset and immunosuppressive molecule MFI values. CD4^+^CD25^+^ T-cells from WT mice showed a positive correlation between CD38 and PD-1 (rS = 0.95, *p* < 0.05), CTLA-4 (rS = 0.95, *p* < 0.05), and IL-10 (rS = 0.6, *p* < 0.05) (Figure 2A). Otherwise, CD38 expression in CD4^+^CD25^−^ T-cells were highly correlated with only CD69 protein (rS = 0.97, *p* < 0.05) (Figure 2B). Regarding B6.MRL-Fas^lpr^/J mice, levels of CD38 expression in CD4^+^CD25^+^ cells exhibited a strong positive correlation with PD-1 (rS = 0.81, *p* < 0.05) and CD69 (rS = 0.84, *p* < 0.05) (Figure 2C), whereas that of CD4^+^CD25^−^ cells showed CD38 correlation with PD-1 (rS = 0.85, *p* < 0.05), CTLA-4 (rS = 0.9, *p* < 0.05), and CD69 (rS = 0.94, *p* < 0.05) (Figure 2D). Together, these data proposed that CD38 expression is consistent with an activated immunosuppressive phenotype.

### 2.3. Reduced CD38^+^ Treg Proportion in B6.MRL-Fas^lpr^/J Mice

**Figure 3:**
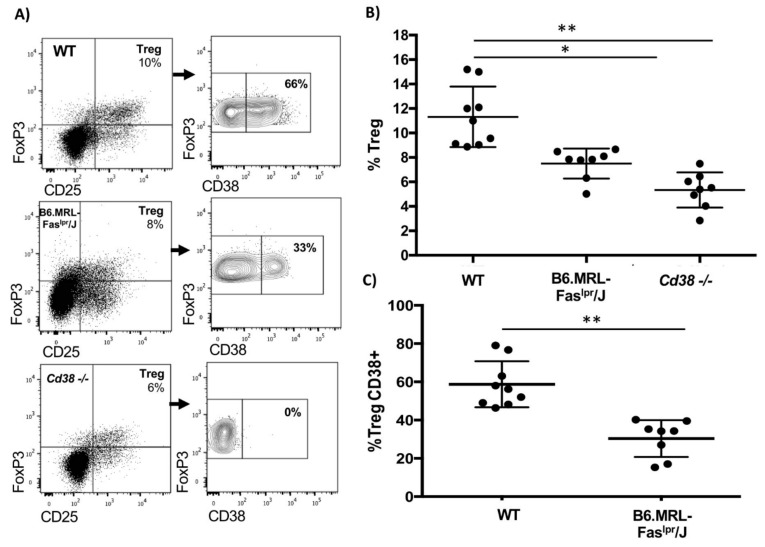
Reduced CD38^+^ Treg levels in B6.MRL-Fas^lpr^/J mice. (**A**) Representative dot plots of the frequency of Treg cells in splenocytes from WT, B6.MRL-Fas^lpr^/J and *CD38-/-*. The dot plots’ right is representative contour plots showing CD38 expression gated on splenic Treg cells from WT (*n* = 9), B6.MRL-Fas^lpr^/J (*n* = 8), and *CD38-/-* mice (*n* = 8); (**B**) scatter dot plot shows mean ± SD of Treg frequency from WT, B6.MRL-Fas^lpr^/J and *CD38-/-* mice. Statistical analysis was performed using one-way ANOVA. Differences between groups were indicated by Tukey post hoc test, where * *p* < 0.001, ** *p* < 0.0001; (**C**) scatter dot plot shows mean ± SD of CD38^+^ Treg frequency from WT and B6. MRL-Fas^lpr^/J mice. Statistical analysis was performed using Student *t*-test, where ** *p* < 0.0001.

Because CD38 correlates with immunosuppressive molecules in T-cells from WT and B6, MRL-Fas^lpr^/J mice, and it could select subsets with suppressive relevance, we wondered if CD38^+^ Treg cells frequency might be impaired in B6.MRL-Fas^lpr^/J mice. First, we identified two main subsets of Treg cells, CD38^+^ and CD38^−^ in WT and B6.MRL-Fas^lpr^/J mice (Figure 3A). Interestingly, the frequency of splenic CD38^+^ Treg cells was significantly decreased from B6.MRL-Fas^lpr^/J in comparison with WT mice (T15 = 5.3, *p* < 0.0001) (Figure 3A,C). Consistently, B6.MRL-Fas^lpr^/J mice showed a decreased proportion of total Treg cells (χ^2^(2) = 17.48, *p* < 0.01) (Figure 3A,B). To figure out CD38 relevance in Treg maintenance, we assessed splenic Treg frequency in *CD38-/-* mice. Compared with WT mice, *CD38-/-* mice showed a lower frequency of Treg cells (χ^2^(2) = 17.48, *p* < 0.001), and non-significative differences were found against B6.MRL-Fas^lpr^/J model (Figure 3B). These results suggested that the development of Treg seems to be affected by the lack of CD38.

**Figure 4:**
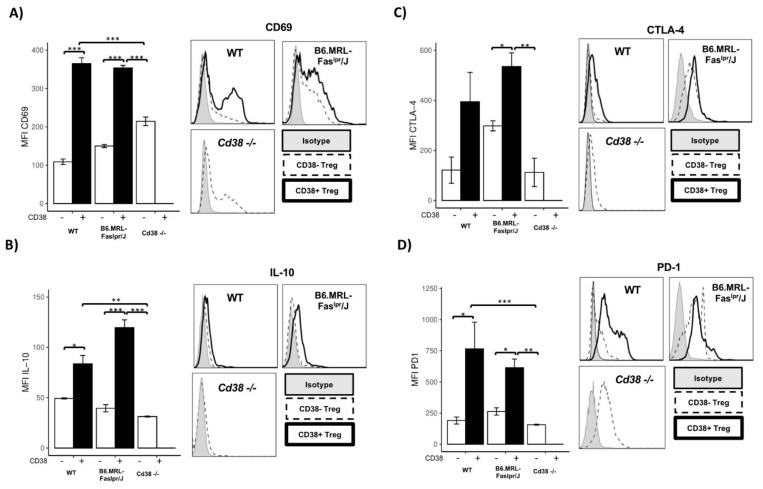
Treg from *CD38-/-* showed a reduction in immunosuppressive molecules. Histograms and bar charts represent the expression of (**A**) CD69, (**B**) IL-10, (**C**) CTLA-4, and (**D**) PD-1 among CD38^−^ and CD38^+^ Treg cells from WT (*n* = 4) and B6.MRL-Fas^lpr^/J (*n* = 4) mice or splenic Treg cells from *CD38-/-* (*n* = 4) mice. Bar charts show mean ± SD. Statistical analysis was performed using two-way ANOVA followed by Tukey test, where *** *p* < 0.0001, ** *p* < 0.001, and * *p* < 0.05.

A previous study in *CD38-/-* mice has revealed some autoimmune disorders in aged mice [22]. Therefore, we asked whether CD38 could be essential to maintain a regulatory phenotype. Therefore, we measured the expression of immunosuppressive molecules in total splenic Treg cells from *CD38-/-* mice, and we compared their expression with CD38^+^ and CD38^−^ Treg cells from WT and B6.MRL-Fas^lpr^/J mice. In the first instance, CD38^+^ Treg cells from WT and B6.MRL-Fas^lpr^/J expressed higher levels of CD69 (Figure 4A), IL-10 (Figure 4B), and PD-1 (Figure 4D) than CD38^−^ Treg cells, while CD38^+^ Treg overexpressed CTLA-4 from B6.MRL-Fas^lpr^/J mice (Figure 4C). Interestingly, when we assessed MFI of CD69, IL-10, CTLA-4, and PD-1 in total splenic Treg from *CD38-/-* mice, we noted a relevant reduction in their expression compared with CD38^+^ Treg from WT and B6.MRL-Fas^lpr^/J mice (Figure 4A–D). Together, results showed that CD38 expression is associated and required to maintain critical suppressive markers.

### 2.4. Reduced Treg Expansion and IL-10 Secretion in Stimulated CD38-/- Splenocytes

**Figure 5:**
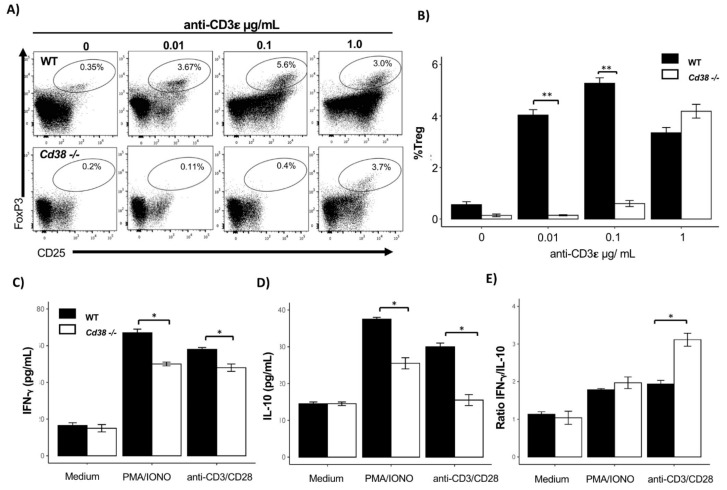
Reduced Treg expansion and IL-10 secretion in stimulated *CD38-/-* splenocytes. Splenocytes from WT (*n* = 3) and *CD38-/-* (*n* = 3) were left unstimulated or stimulated with indicated concentrations of anti-CD3 for 72 h. Cells were stained with anti-CD4 and anti-CD25, followed by intracellular staining for Foxp3. (**A**) Representative dot plots of Treg gated according to CD25 and FoxP3 expression. Numbers in the oval indicate the percentage of Treg cells after stimulation; (**B**) percentage (mean ± SEM) of Treg cells obtained from the culture; (**C**) concentrations (mean ± SEM) of IFN-γ and (**D**) IL-10 release in splenocyte-culture supernatant upon in vitro stimulation with anti-CD3/CD28 + rhIL-2 for 72 h; (**E**) IFN-γ/IL-10 ratio. Statistical analysis was performed using two-way ANOVA followed by Tukey test, where ** *p* < 0.001, and * *p* < 0.05.

Given that TCR signals play a crucial role in Treg cell maintenance and proliferation [33,34], we examined whether the lack of CD38 could impair the proliferative ability of Treg cells stimulated via CD3. Total splenocytes from WT and *CD38-/-* mice were left unstimulated or stimulated with different concentrations of anti-CD3 for 72 h, as described in materials and methods. After cell surface staining for CD4 and CD25, cultured cells were intracellularly stained for Foxp3. As depicted in Figure 5A,B, Treg cells from *CD38-/-* mice showed significantly reduced frequency than WT mice specially at 0.01 (T_4_ = 16.07, *p* < 0.0001) and 0.1 µg/mL (T_4_ = 20.7, *p* < 0.0001) of anti-CD3.

Splenocytes stimulated with anti-CD3/CD28 + rhIL2 or mitogens such as phorbol myristate acetate plus ionomycin (PMA/IONO) promote the release of pro-inflammatory and anti-inflammatory cytokines such as IFN-γ and IL-10, respectively [35,36]. A good balance between the pro-inflammatory IFN-γ and anti-inflammatory IL-10 is an indicator of reciprocal immune regulation [37,38]. Therefore, to understand the role of CD38 in this phenomenon, we analyzed the ability of the expanded splenocytes from *CD38-/-* mice to produce IFN-γ that might either enhance or abrogate Treg function. Analysis of culture supernatants of *CD38-/-* splenocytes stimulated with anti-CD3/CD28 + rhIL2 and PMA/IONO showed a reduced concentration of IFN-γ and IL-10 compared to supernatants of WT splenocytes (Figure 5C,D). In addition, the ratio of IFN-γ/IL-10 was detected to be significantly higher in supernatants from *CD38-/-* stimulated splenocytes, compared with WT splenocytes (Figure 5E).

## 3. Discussion

An unprecedented number of studies performed in SLE patients have demonstrated that CD38 is widely expressed in T-cells, showing that CD25^+^CD38^+^ T-cells are increased regarding healthy controls [15,16,17,18,19]. It has been proposed that the increase in CD25^+^CD38^+^ T-cells in SLE disease could be indicative of persistent T-cell activation [19]. However, the contribution of CD38 to the pathology of SLE remains unclear. Since CD38 is a transmembrane glycoprotein widely expressed in several immune cells, its function during SLE development might involve different pathways and effector functions according to the cell type where it is expressed.

Previous reports have described that CD38 expression might identify CD4^+^ T-cell subsets with immunomodulatory properties [39]; thus, we hypothesize CD38 expression in T-cells from SLE patients can mediate disease development through regulatory activity. To assess it, we used B6.MRL-Fas^lpr^/J lupus murine model, which is widely used to understand autoimmune disease’s physiological and pathological processes [40,41]. Thus, first, we analyzed and compared regulatory phenotype through FoxP3 expression in different subsets of CD4^+^ T-cells divided according to CD25 and CD38 expression in WT and B6.MRL-Fas^lpr^/J mice. We identified three different levels of CD38 expression (CD38^high^, CD38^mid^, CD38^low^) within CD4^+^CD25^+^ T-cells and CD4^+^CD25^−^ T-cells. In this study, we describe, for the first time, a positive correlation between CD38 levels and Foxp3 frequency expression in both CD25^+^ and CD25^−^ subsets, highlighting a higher correlation in B6.MRL-Fas^lpr^/J than in WT mice (Figure 1C). Although there is no evidence suggesting FoxP3 as a transcription factor of the CD38 gene, it is widely known that all-trans retinoic acid (ATRA) increases CD38 and FoxP3 expression [42]. The first intron of CD38 contains a binding site for the canonical ATRA receptor, the transcription factor RARα [43]. Moreover, ATRA acts as an inducer of Treg response in CD4^+^ T-cells via demethylation of the FOXP3 promoter and activation of FOXP3 expression [44,45]. An environment enriched with ATRA, mainly produced by CD103^+^ dendritic cells during antigen presentation, might increase Treg differentiation and CD38 expression.

CD4^+^CD25^−^ T-cells have been proposed, with the potential to switch spontaneously in the periphery into Treg cells [46,47]. Patients with active SLE have shown an increased amount of CD4^+^CD25^−^FoxP3^+^ T-cells correlating with lupus nephritis [48,49]. CD25^−^ cells from WT and B6.MRL-Fas^lpr^/J mice also increased immunosuppressive molecules such as CTLA-4 and PD-1, as well as CD69 marker. Although CD69 is generally associated as the earliest activation cell surface marker on leukocytes, it has been reported that CD25^−^FoxP3^−^CD69^+^ cells from a tumor-bearing mouse can inhibit CD4^+^ T-cell proliferation in vitro and in vivo [50]. We found a positive correlation between CD38 and CD69 in CD4^+^CD25^−^ T-cells from WT, where we also found a reduced amount of FoxP3^+^ cells. Likewise, CD4^+^CD25^−^ T-cells from B6.MRL-Fas^lpr^/J showed a positive correlation with CD69 and PD-1 and CTLA-4, exhibiting a higher amount of FoxP3 cells than WT mice. Considering these data, we propose that, due to a lack of regulation in B6.MRL-Fas^lpr^/J mice, CD25^−^CD38^high^ T-cells could identify subsets with a high potential to be converted into Treg cells. However, the decreased expression of CD25 and the impaired ability to produce IL-2 by B6.MRL-Fas^lpr^/J mice [51,52] could avoid in vivo Treg induction, increasing cell death probability. Further studies are necessary to understand this mechanism in mice and SLE patients.

It is well known that CD4^+^CD25^+^ T-cells are essential in maintaining peripheral tolerance and controlling autoreactive T-cells in humans and mice due to their higher levels of FoxP3 expression [53]. Here, we found a positive correlation in WT CD4^+^CD25^+^ T-cells between CD38 and PD-1, CTLA-4, and IL-10, while in B6.MRL-Fas^lpr^/J, CD38 only correlated with PD-1 and CD69. Thus, it can be suggested that CD38 overexpression could define Treg cells with a highly suppressive activity that might decrease in B6.MRL-Fas^lpr^/J mice. When we evaluated CD38^+^ Treg cells from B6.MRL-Faslpr/J mice, we found them dramatically decreased compared with WT CD38^+^ Treg cells. Interestingly, CD38^+^ Treg cells overexpressed immunosuppressive markers compared to CD38- Treg cells in the same way as CD38^+^ Treg from WT mice. Additionally, Treg from *CD38-/-* downregulated immunosuppressive molecules relative CD38^+^ Treg cells from WT and B6.MRL-Fas^lpr^/J mice, suggesting that CD38 is relevant to maintain a suppressive phenotype. Previously, CD38^+^ Treg cells have been defined as a highly suppressive subset in patients with multiple myeloma [31,32] and have shown an increased ability to inhibit immune response [42]. We have suggested that a reduced CD38^+^ Treg could indicate Treg loss with suppressive function in B6.MRL-Fas^lpr^/J mice. Thus, we proposed that CD38 expression in Treg might identify functional Treg cells, highlighting that a higher proportion of CD38^+^ Treg cells could revert or prevent SLE development in B6.MRL-Fas^lpr^/J mice.

CD38 is a transmembrane glycoprotein with ectoenzyme and receptor activity, such as ectoenzyme CD38, the principal NAD^+^ consumer and regulator in several conditions [54]. Previous reports have established that NAD^+^ conversion through CD38 may generate adenosine (ADO), which has been associated with inducing FoxP3 expression and promote CTLA-4 expression, significantly increasing immunoregulatory activity [55,56]. Furthermore, it has been shown that CD38 absence in murine models with autoimmunity enhances the development of the disease. More recently, our laboratory found that only CD38 deficiency in a WT background induced an autoimmune phenotype such as SLE [22]. In this work, we confirmed a Treg deficiency in *CD38-/-* mice, as previous results were reported [42,57]. Explanation to this loss of Treg cells in *CD38-/-* mice include the role of CD38 in removing NAD^+^ and preventing its binding to the mono-ADP-ribosyltransferase ART2, an enzyme that ribosylates P2X7 and induces apoptosis of CD4^+^CD25^+^ cells [57,58,59].

We also reported that Treg cells from *CD38-/-* mice showed an impaired expansion ability when stimulated via CD3. Even though there is no evidence of CD38 signaling in Treg, several studies have proposed that CD38 signaling in T-cells is initiated within a subset of membrane rafts [60], activating a transduction pathway that includes ζ-associated protein-70, CD3ɛ, phospholipase C-γ, Raf-1/mitogen-activated protein kinase, and calcium mobilization [61,62,63]. Additionally, CD38 signaling requires a T-cell receptor (TCR)/CD3 receptor complex, suggesting a functional association with TCR signal transduction machinery [61,62,63]. Remarkably, we proposed that CD38 also participates in the TCR signaling pathway to maintain Treg cells.

It is known that induction of IFN-γ often occurs together with anti-inflammatory cytokines such as IL-10 as a mechanism to reduce the uncontrolled production of inflammatory cytokines [37,38]. Here, we found the ratio IFN-γ/IL-10 decreased in *CD38-/-* mice than WT. These findings are in contradiction with data previously reported by Burlock and cols., which mentioned that CD38 deficiency increased IL-10 producing B cells and reduced IFN-α production in the peritoneal cavity [64] and with Domínguez and cols., who found no differences in IL-10-producing B cells between *CD38-/-* and WT mice [22]. In this work, we are evaluating secretion of IL-10 from splenocytes stimulated with anti-CD3 and PMA/IONO stimulus for 72 h, identifying IL-10 released after the proliferation of T-cells or total splenocytes, respectively. In contrast, they identified mainly intracellular IL-10 in B10 cells by shorter periods of stimulation with LPS. Otherwise, CD38 blocking with Isatuximab in Treg cells from Myeloma Multiple patients has demonstrated a reduced IL-10 expression and a higher amount of IFN-γ expression in CD8^+^ T-cells [31]. However, mechanisms of IL-10 secretion are different in each cellular type; thus, we have to perform additional experiments to elucidate if Treg from *CD38-/-* loss ability to produce IL-10, an important mechanism to maintain tolerance and avoid the development of autoimmune disease in *CD38-/-* mice of advanced age.

As a whole, our data demonstrate that CD38 correlates with FoxP3 expression and immunosuppressive markers in a murine model of SLE. Thus, we proposed that CD38 expression is vital to maintain Treg cells homeostasis and to prevent autoimmune disease. Future work will focus on characterizing the absence of CD38 in B6.MRL-Fas^lpr^/J mice and its effect on the immune regulation to clarify controversial data supporting both beneficial and pathogenic roles for CD38 in SLE disease.

Furthermore, additional studies must be performed to understand the interaction between CD38 receptor and TCR to induce functional Treg cells. Nevertheless, the present findings might help to suggest new therapeutic strategies in SLE.

## 4. Materials and Methods

### 4.1. Mice

*C57BL/6* wild-type (WT), *C57BL/6 CD38-/- (CD38-/-*), and C57BL/6-MRL-Fas^lpr^/J (B6.MRL-Fas^lpr^/J) mice were purchased from The Jackson Laboratory (Bar Harbor, ME, USA). According to national regulations, the mice were bred by strain to obtain littermates in the Universidad Veracruzana animal facility. Male mice between 8 and 12 weeks old were used for experiments. All mice were maintained in specific pathogen-free conditions with a 1:1 light-dark cycle. Food and water were provided ad libitum.

Institutional Animal Care approved all procedures for animals (No. 2018-0004). In addition, the authors adhere to the guidelines established by Mexico (Norma Oficial Mexicana NOM-062-ZOO-1999).

### 4.2. Isolation of Splenocytes

Mice were euthanized by cervical dislocation, and their spleens were excised. Splenocytes were dissociated from the connective tissue capsule by gently pressing the organ through a 200-mesh sterile metal sieve. Erythrocytes were depleted with 0.85% ammonium chloride solution. Cells were washed once with phosphate-buffered saline (PBS 1×) and resuspended in PBS 1× (Corning, Manassas, VA, USA).

### 4.3. Flow Cytometry

For surface staining, cells were stained with anti-CD3 PerCP-Cy5.5, anti-CD4 APC, anti-CD25 APC-Cy7, anti-CD69 Pe-Cy7, anti-PD-1 BV421, and anti-CD38 Alexa Fluor 488 (all from BD Biosciences, San Diego, CA, USA). Cells were then incubated for 15 minutes under room temperature and were fixed with 1% formaldehyde for 15 minutes. For intracellular staining, cells were permeabilized (Cytofix/Cytoperm TM Fixation/Permeabilization Solution Kit, BD Biosciences) for 40 minutes and stained with anti-FoxP3 Alexa Fluor 488, anti-CTLA-4 BV605, and anti-IL-10 APC. All the samples were analyzed using an LSRFortessa flow cytometer (Becton-Dickinson, San Jose, CA, USA) and analyzed by FlowJo software (FlowJo, Tree Star, Ashland, OR, USA).

### 4.4. Expansion Treg Assay

Cells (1 × 10^6^ cells/mL) were set up in 24-well tissue culture plate wells (Corning, Manassas, VA, USA) in a RPMI-1640 medium containing 10% fetal bovine serum, 100 µmol/L non-essential amino acids (Sigma Chemicals, St. Louis, MO, USA), 1 mmol/L sodium pyruvate (Gibco), 0.2 mg/mL penicillin (Gibco), 0.5 mg/mL streptomycin (Gibco), HEPES 10 mmol/L, and 5.5 × 10^−5^ mol/L β-mercaptoethanol (Gibco). Afterward, cells were left unstimulated or stimulated with an anti-CD3ε Ab (BD Biosciences, San Diego, CA, USA) at the indicated concentrations. The plates were incubated at 37 °C in the presence of 5% CO_2_. After incubation, the cells were harvested and washed with PBS. Finally, they were stained with anti-CD4 APC, anti-CD25 APC-Cy7, and anti-FoxP3 PE, as we described above.

### 4.5. IFN-γ and IL-10 Detection

Splenocytes from WT and *CD38-/-* mice were left unstimulated or stimulated with 1 µg/mL of anti-CD3ε Ab (BD Biosciences, San Diego, CA, USA), 1 µg/mL of anti-CD28 Ab (BD Biosciences, San Diego, CA, USA), and 200U rhIL-2 (Sigma Aldrich) or Phorbol Myristate Acetate (50 ng/mL) plus Ionomycin (500 ng/mL) for 72 h. According to the manufacturer’s protocol, supernatant concentrations of IFN-γ and IL-10 were evaluated by Mouse ELISA MAXTM DELUXE Set (Biolegend, San Diego, CA, USA).

### 4.6. Statistical Analysis

Shapiro–Wilk tests were used to test the normality of the data distributions. Correlation tests were performed between CD38 and FoxP3, CTLA-4, CD69, PD1, and IL-10 using the non-parametric Spearman test, where an “r” between 0.8 and 1 indicated a high positive correlation; from 0.4 to 0.79, a mean positive correlation, and from 0.01 to 0.39, a low positive correlation was considered. The results were statistically significant when the value of *p* < 0.05. Treg and Treg CD38^+^ frequency was performed using the non-parametric Kruskal–Wallis and t-Student tests, respectively, where a *p* < 0.05 was considered statistically significant. Finally, the cell culture results were evaluated by a two-way analysis of variance (ANOVA). Tukey post hoc test was used for multiple comparisons, as indicated. Statistical significance was determined with *p* < 0.05. All data were analyzed using the statistical program RStudio 1.3.1093, © 2009–2020 (RStudio, PBC Inc., Boston, MA, USA).

## References

[1] Fava A., Petri M. (2019). Systemic Lupus Erythematosus: Diagnosis and Clinical Management. J. Autoimmun..

[2] Nusbaum J.S., Mirza I., Shum J., Freilich R.W., Cohen R.E., Pillinger M.H., Izmirly P.M., Buyon J.P. (2020). Sex Differences in Systemic Lupus Erythematosus: Epidemiology, Clinical Considerations, and Disease Pathogenesis. Mayo Clin. Proc..

[3] Karagianni P., Tzioufas A.G. (2019). Epigenetic Perspectives on Systemic Autoimmune Disease. J. Autoimmun..

[4] Yeoh S.-A., Dias S.S., Isenberg D.A. (2018). Advances in Systemic Lupus Erythematosus. Medicine.

[5] Gray-McGuire C., Moser K.L., Gaffney P.M., Kelly J., Yu H., Olson J.M., Jedrey C.M., Jacobs K.B., Kimberly R.P., Neas B.R. (2000). Genome Scan of Human Systemic Lupus Erythematosus by Regression Modeling: Evidence of Linkage and Epistasis. Am. J. Hum. Genet..

[6] Lindqvist A.K., Alarcón-Riquelme M.E. (1999). The Genetics of Systemic Lupus Erythematosus. Scand. J. Immunol..

[7] Nakagawara K., Mori M., Takasawa S., Nata K., Takamura T., Berlova A., Tohgo A., Karasawa T., Yonekura H., Takeuchi T. (1995). Assignment of CD38, the Gene Encoding Human Leukocyte Antigen CD38 (ADP-Ribosyl Cyclase/Cyclic ADP-Ribose Hydrolase), to Chromosome 4p15. Cytogenet. Genome Res..

[8] Funaro A., Spagnoli G.C., Ausiello C.M., Alessio M., Roggero S., Delia D., Zaccolo M., Malavasi F. (1990). Involvement of the Multilineage CD38 Molecule in a Unique Pathway of Cell Activation and Proliferation. J. Immunol..

[9] States D.J., Walseth T.F., Lee H.C. (1992). Similarities in Amino Acid Sequences of Aplysia ADP-Ribosyl Cyclase and Human Lymphocyte Antigen CD38. Trends Biochem. Sci..

[10] Wei W., Graeff R., Yue J. (2014). Roles and Mechanisms of the CD38/Cyclic Adenosine Diphosphate Ribose/Ca^2+^ Signaling Pathway. World J. Biol. Chem..

[11] Santos-Argumedo L., Teixera C., Preece G., Kirkham P., Parkhouse R.M. (1993). A B Lymphocyte Surface Molecule Mediating Activation and Protection from Apoptosis via Calcium Channels. J. Immunol..

[12] Partida-Sánchez S., Goodrich S., Kusser K., Oppenheimer N., Randall T.D., Lund F.E. (2004). Regulation of Dendritic Cell Trafficking by the ADP-Ribosyl Cyclase CD38: Impact on the Development of Humoral Immunity. Immunity.

[13] Lund F.E., Muller-Steffner H., Romero-Ramirez H., Moreno-García M.E., Partida-Sánchez S., Makris M., Oppenheimer N.J., Santos-Argumedo L., Schuber F. (2006). CD38 Induces Apoptosis of a Murine Pro-B Leukemic Cell Line by a Tyrosine Kinase-Dependent but ADP-Ribosyl Cyclase- and NAD Glycohydrolase-Independent Mechanism. Int. Immunol..

[14] Rodríguez-Alba J.C., Moreno-García M.E., Sandoval-Montes C., Rosales-Garcia V.H., Santos-Argumedo L. (2008). CD38 Induces Differentiation of Immature Transitional 2 B Lymphocytes in the Spleen. Blood.

[15] Alcocer-Varela J., Alarcón-Riquelme M., Laffón A., Sánchez-Madrid F., Alarcón-Segovia D. (1991). Activation Markers on Peripheral Blood T-cells from Patients with Active or Inactive Systemic Lupus Erythematosus. Correlation with Proliferative Responses and Production of IL-2. J. Autoimmun..

[16] al-Janadi M., Raziuddin S. (1993). B Cell Hyperactivity Is a Function of T-cell Derived Cytokines in Systemic Lupus Erythematosus. J. Rheumatol..

[17] Pavón E.J., Muñoz P., Navarro M.-C., Raya-Alvarez E., Callejas-Rubio J.-L., Navarro-Pelayo F., Ortego-Centeno N., Sancho J., Zubiaur M. (2006). Increased Association of CD38 with Lipid Rafts in T-cells from Patients with Systemic Lupus Erythematosus and in Activated Normal T-cells. Mol. Immunol..

[18] Spronk P.E., Horst G., Van Der Gun B.T., Limburg P.C., Kallenberg C.G. (1996). Anti-DsDNA Production Coincides with Concurrent B and T-cell Activation during Development of Active Disease in Systemic Lupus Erythematosus (SLE). Clin. Exp. Immunol..

[19] Pavón E.J., Zumaquero E., Rosal-Vela A., Khoo K.-M., Cerezo-Wallis D., García-Rodríguez S., Carrascal M., Abian J., Graeff R., Callejas-Rubio J.-L. (2013). Increased CD38 Expression in T-cells and Circulating Anti-CD38 IgG Autoantibodies Differentially Correlate with Distinct Cytokine Profiles and Disease Activity in Systemic Lupus Erythematosus Patients. Cytokine.

[20] Chen J., Chen Y.-G., Reifsnyder P.C., Schott W.H., Lee C.-H., Osborne M., Scheuplein F., Haag F., Koch-Nolte F., Serreze D.V. (2006). Targeted Disruption of CD38 Accelerates Autoimmune Diabetes in NOD/Lt Mice by Enhancing Autoimmunity in an ADP-Ribosyltransferase 2-Dependent Fashion. J. Immunol..

[21] Viegas M.S., Silva T., Monteiro M.M., do Carmo A., Martins T.C. (2011). Knocking out of CD38 Accelerates Development of a Lupus-like Disease in Lpr Mice. Rheumatology.

[22] Domínguez-Pantoja M., López-Herrera G., Romero-Ramírez H., Santos-Argumedo L., Chávez-Rueda A.K., Hernández-Cueto Á., Flores-Muñoz M., Rodríguez-Alba J.C. (2018). CD38 Protein Deficiency Induces Autoimmune Characteristics and Its Activation Enhances IL-10 Production by Regulatory B Cells. Scand. J. Immunol..

[23] Horwitz D.A. (2008). Regulatory T-cells in Systemic Lupus Erythematosus: Past, Present and Future. Arthritis Res. Ther..

[24] Sakaguchi S., Sakaguchi N., Asano M., Itoh M., Toda M. (1995). Immunologic Self-Tolerance Maintained by Activated T-cells Expressing IL-2 Receptor Alpha-Chains (CD25). Breakdown of a Single Mechanism of Self-Tolerance Causes Various Autoimmune Diseases. J. Immunol..

[25] Sakaguchi S., Yamaguchi T., Nomura T., Ono M. (2008). Regulatory T-cells and Immune Tolerance. Cell.

[26] Parry R.V., Chemnitz J.M., Frauwirth K.A., Lanfranco A.R., Braunstein I., Kobayashi S.V., Linsley P.S., Thompson C.B., Riley J.L. (2005). CTLA-4 and PD-1 Receptors Inhibit T-Cell Activation by Distinct Mechanisms. Mol. Cell. Biol..

[27] Waterhouse P., Penninger J.M., Timms E., Wakeham A., Shahinian A., Lee K.P., Thompson C.B., Griesser H., Mak T.W. (1995). Lymphoproliferative Disorders with Early Lethality in Mice Deficient in Ctla-4. Science.

[28] Taylor A., Verhagen J., Blaser K., Akdis M., Akdis C.A. (2006). Mechanisms of Immune Suppression by Interleukin-10 and Transforming Growth Factor-Beta: The Role of T Regulatory Cells. Immunology.

[29] Wei F., Zhong S., Ma Z., Kong H., Medvec A., Ahmed R., Freeman G.J., Krogsgaard M., Riley J.L. (2013). Strength of PD-1 Signaling Differentially Affects T-Cell Effector Functions. Proc. Natl. Acad. Sci. USA.

[30] Fontenot J.D., Gavin M.A., Rudensky A.Y. (2003). Foxp3 Programs the Development and Function of CD4^+^CD25^+^ Regulatory T-cells. Nat. Immunol..

[31] Feng X., Zhang L., Acharya C., An G., Wen K., Qiu L., Munshi N.C., Tai Y.-T., Anderson K.C. (2017). Targeting CD38 Suppresses Induction and Function of T Regulatory Cells to Mitigate Immunosuppression in Multiple Myeloma. Clin. Cancer Res..

[32] Krejcik J., Casneuf T., Nijhof I.S., Verbist B., Bald J., Plesner T., Syed K., Liu K., van de Donk N.W.C.J., Weiss B.M. (2016). Daratumumab Depletes CD38^+^ Immune Regulatory Cells, Promotes T-Cell Expansion, and Skews T-Cell Repertoire in Multiple Myeloma. Blood.

[33] Li M.O., Rudensky A.Y. (2016). T-cell Receptor Signaling in the Control of Regulatory T-cell Differentiation and Function. Nat. Rev. Immunol..

[34] Chen Y., Shen S., Gorentla B.K., Gao J., Zhong X.-P. (2012). Murine Regulatory T-cells Contain Hyperproliferative and Death-Prone Subsets with Differential ICOS Expression. J. Immunol..

[35] Liang Y., Cucchetti M., Roncagalli R., Yokosuka T., Malzac A., Bertosio E., Imbert J., Nijman I.J., Suchanek M., Saito T. (2013). The Lymphoid Lineage—Specific Actin-Uncapping Protein Rltpr Is Essential for Costimulation via CD28 and the Development of Regulatory T-cells. Nat. Immunol..

[36] Schwarz M., Majdic O., Knapp W., Holter W. (1995). High-Level IL-10 Production by Monoclonal Antibody-Stimulated Human T-cells. Immunology.

[37] Yanagawa Y., Iwabuchi K., Onoé K. (2009). Co-Operative Action of Interleukin-10 and Interferon-γ to Regulate Dendritic Cell Functions. Immunology.

[38] Saraiva M., O’Garra A. (2010). The Regulation of IL-10 Production by Immune Cells. Nat. Rev. Immunol..

[39] Read S., Mauze S., Asseman C., Bean A., Coffman R., Powrie F. (1998). CD38^+^ CD45RBlow CD4^+^ T-cells: A Population of T-cells with Immune Regulatory Activities in Vitro. Eur. J. Immunol..

[40] Cohen P.L., Eisenberg R.A. (1991). Lpr and Gld: Single Gene Models of Systemic Autoimmunity and Lymphoproliferative Disease. Annu. Rev. Immunol..

[41] van Nieuwenhuijze A.E., Cauwe B., Klatt D., Humblet-Baron S., Liston A. (2015). Lpr-Induced Systemic Autoimmunity Is Unaffected by MasT-cell Deficiency. Immunol. Cell Biol..

[42] Patton D.T., Wilson M.D., Rowan W.C., Soond D.R., Okkenhaug K. (2011). The PI3K P110δ Regulates Expression of CD38 on Regulatory T-cells. PLoS ONE.

[43] Drach J., McQueen T., Engel H., Andreeff M., Robertson K.A., Collins S.J., Malavasi F., Mehta K. (1994). Retinoic Acid-Induced Expression of CD38 Antigen in Myeloid Cells Is Mediated through Retinoic Acid Receptor-Alpha. Cancer Res..

[44] Mucida D., Pino-Lagos K., Kim G., Nowak E., Benson M.J., Kronenberg M., Noelle R.J., Cheroutre H. (2009). Retinoic Acid Can Directly Promote TGF-Beta-Mediated Foxp3(+) Treg Cell Conversion of Naive T-cells. Immunity.

[45] Sun X., Xiao Y., Zeng Z., Shi Y., Tang B., Long H., Kanekura T., Wang J., Wu H., Zhao M. (2018). All-Trans Retinoic Acid Induces CD4^+^CD25^+^FOXP3^+^ Regulatory T-cells by Increasing FOXP3 Demethylation in Systemic Sclerosis CD4^+^ T-cells. J. Immunol. Res..

[46] Yan B., Liu Y. (2009). The Nature of Increased Circulating CD4^+^CD25^−^Foxp3^+^ T-cells in Patients with Systemic Lupus Erythematosus: A Novel Hypothesis. Open Rheumatol. J..

[47] Zelenay S., Lopes-Carvalho T., Caramalho I., Moraes-Fontes M.F., Rebelo M., Demengeot J. (2005). Foxp3^+^ CD25- CD4 T-cells Constitute a Reservoir of Committed Regulatory Cells That Regain CD25 Expression upon Homeostatic Expansion. Proc. Natl. Acad. Sci. USA.

[48] Bonelli M., Göschl L., Blüml S., Karonitsch T., Steiner C.-W., Steiner G., Smolen J.S., Scheinecker C. (2014). CD4^+^CD25^−^Foxp3^+^ T-cells: A Marker for Lupus Nephritis?. Arthritis Res. Ther..

[49] Ferreira R.C., Simons H.Z., Thompson W.S., Rainbow D.B., Yang X., Cutler A.J., Oliveira J., Castro Dopico X., Smyth D.J., Savinykh N. (2017). Cells with Treg-Specific FOXP3 Demethylation but Low CD25 Are Prevalent in Autoimmunity. J. Autoimmun..

[50] Han Y., Guo Q., Zhang M., Chen Z., Cao X. (2009). CD69^+^ CD4^+^ CD25^−^ T-cells, a New Subset of Regulatory T-cells, Suppress T-cell Proliferation through Membrane-Bound TGF-Beta 1. J. Immunol..

[51] Altman A., Theofilopoulos A.N., Weiner R., Katz D.H., Dixon F.J. (1981). Analysis of T-cell Function in Autoimmune Murine Strains. Defects in Production and Responsiveness to Interleukin 2. J. Exp. Med..

[52] Wofsy D., Murphy E.D., Roths J.B., Dauphinée M.J., Kipper S.B., Talal N. (1981). Deficient Interleukin 2 Activity in MRL/Mp and C57BL/6J Mice Bearing the Lpr Gene. J. Exp. Med..

[53] Zhang L., Zhao Y. (2007). The Regulation of Foxp3 Expression in Regulatory CD4(+)CD25(+)T-cells: Multiple Pathways on the Road. J. Cell. Physiol..

[54] Horenstein A.L., Chillemi A., Zaccarello G., Bruzzone S., Quarona V., Zito A., Serra S., Malavasi F. (2013). A CD38/CD203a/CD73 Ectoenzymatic Pathway Independent of CD39 Drives a Novel Adenosinergic Loop in Human T Lymphocytes. Oncoimmunology.

[55] Bao R., Hou J., Li Y., Bian J., Deng X., Zhu X., Yang T. (2016). Adenosine Promotes Foxp3 Expression in Treg Cells in Sepsis Model by Activating JNK/AP-1 Pathway. Am. J. Transl. Res..

[56] Ohta A., Kini R., Ohta A., Subramanian M., Madasu M., Sitkovsky M. (2012). The Development and Immunosuppressive Functions of CD4(+) CD25(+) FoxP3(+) Regulatory T-cells Are under Influence of the Adenosine-A2A Adenosine Receptor Pathway. Front. Immunol..

[57] Hubert S., Rissiek B., Klages K., Huehn J., Sparwasser T., Haag F., Koch-Nolte F., Boyer O., Seman M., Adriouch S. (2010). Extracellular NAD^+^ Shapes the Foxp3^+^ Regulatory T-cell Compartment through the ART2–P2X7 Pathway. J. Exp. Med..

[58] Aswad F., Kawamura H., Dennert G. (2005). High Sensitivity of CD4^+^CD25^+^ Regulatory T-cells to Extracellular Metabolites Nicotinamide Adenine Dinucleotide and ATP: A Role for P2X7 Receptors. J. Immunol..

[59] Aswad F., Dennert G. (2006). P2X7 Receptor Expression Levels Determine Lethal Effects of a Purine Based Danger Signal in T Lymphocytes. Cell. Immunol..

[60] Muñoz P., Navarro M.-C., Pavón E.J., Salmerón J., Malavasi F., Sancho J., Zubiaur M. (2003). CD38 Signaling in T-cells Is Initiated within a Subset of Membrane Rafts Containing Lck and the CD3-Zeta Subunit of the T-cell Antigen Receptor. J. Biol. Chem..

[61] Zubiaur M., Izquierdo M., Terhorst C., Malavasi F., Sancho J. (1997). CD38 Ligation Results in Activation of the Raf-1/Mitogen-Activated Protein Kinase and the CD3-Zeta/Zeta-Associated Protein-70 Signaling Pathways in Jurkat T Lymphocytes. J. Immunol..

[62] Zubiaur M., Guirado M., Terhorst C., Malavasi F., Sancho J. (1999). The CD3-Gamma Delta Epsilon Transducing Module Mediates CD38-Induced Protein-Tyrosine Kinase and Mitogen-Activated Protein Kinase Activation in Jurkat T-cells. J. Biol. Chem..

[63] Zubiaur M., Fernández O., Ferrero E., Salmerón J., Malissen B., Malavasi F., Sancho J. (2002). CD38 Is Associated with Lipid Rafts and upon Receptor Stimulation Leads to Akt/Protein Kinase B and Erk Activation in the Absence of the CD3-Zeta Immune Receptor Tyrosine-Based Activation Motifs. J. Biol. Chem..

[64] Burlock B., Richardson G., García-Rodríguez S., Guerrero S., Zubiaur M., Sancho J. (2018). The Role of CD38 on the Function of Regulatory B Cells in a Murine Model of Lupus. Int. J. Mol. Sci..

